# Increased immune marker variance in a population of invasive birds

**DOI:** 10.1038/s41598-020-78427-7

**Published:** 2020-12-10

**Authors:** Hanna Prüter, Mathias Franz, Sönke Twietmeyer, Niklas Böhm, Gudrun Middendorff, Ruben Portas, Jörg Melzheimer, Holger Kolberg, Georg von Samson-Himmelstjerna, Alex D. Greenwood, Dörte Lüschow, Kristin Mühldorfer, Gábor Árpád Czirják

**Affiliations:** 1 Department of Wildlife Diseases, Leibniz Institute for Zoo and Wildlife Research, Alfred-Kowalke-Straße 17, 10315 Berlin, Germany; 2 Department of Research and Documentation, Eifel National Park, Urftseestraße 43, 53937 Schleiden-Gemünd, Germany; 3 FÖA Landschaftsplanung GmbH, Auf der Redoute 12, 54296 Trier, Germany; 4 Namibia Bird Club, Windhoek, Namibia; 5 Department of Evolutionary Ecology, Leibniz Institute for Zoo and Wildlife Research, Alfred-Kowalke-Straße 17, 10315 Berlin, Germany; 6 Ministry of Environment and Tourism, Private Bag, 13306 Windhoek, Namibia; 7 Institute for Parasitology and Tropical Veterinary Medicine, Freie Universität Berlin, Robert-von-Ostertag-Str. 7-13, 14163 Berlin, Germany; 8 Department of Veterinary Medicine, Freie Universität Berlin, 14163 Berlin, Germany; 9 Institute of Poultry Diseases, Freie Universität Berlin, Königsweg 63, 14163 Berlin, Germany

**Keywords:** Ecology, Evolution, Immunology, Microbiology

## Abstract

Immunity and parasites have been linked to the success of invasive species. Especially lower parasite burden in invasive populations has been suggested to enable a general downregulation of immune investment (Enemy Release and Evolution of Increased Competitive Ability Hypotheses). Simultaneously, keeping high immune competence towards potentially newly acquired parasites in the invasive range is essential to allow population growth. To investigate the variation of immune effectors of invasive species, we compared the mean and variance of multiple immune effectors in the context of parasite prevalence in an invasive and a native Egyptian goose (*Alopochen aegyptiacus*) population. Three of ten immune effectors measured showed higher variance in the invasive population. Mean levels were higher in the invasive population for three effectors but lower for eosinophil granulocytes. Parasite prevalence depended on the parasite taxa investigated. We suggest that variation of specific immune effectors, which may be important for invasion success, may lead to higher variance and enable invasive species to reduce the overall physiological cost of immunity while maintaining the ability to efficiently defend against novel parasites encountered.

## Introduction

Invasive species are major threats to global biodiversity^1,2^. Moreover, they may affect animal and public health by playing important epidemiological roles in spreading and maintaining several micro- and macro-parasites (from here on designated ‘parasites’)^3^. With increasing globalisation and increasing biodiversity loss, studying the underlying mechanisms which promote species to become invasive, is essential for risk assessment, species conservation efforts and public health^4^.

Although, the introduction of exotic, neozootic species to novel habitats is common, only few become true invaders. After introduction, neozootic species need to become established and spread to successfully invade the new region^5^. Environmental factors, available resources and natural enemies (i.e. predators and parasites) are important factors determining the population growth rate and invasion success of such species^6^. Resources are typically limited and must be allocated among different life history traits or stages, such as individual growth, dispersion, reproduction and immunity^7,8^. Allocating resources from immunity to other physiological processes, such as reproduction, has been suggested to increase invasion success^9^.

Allocating resources away from immunity is especially beneficial when parasite burden is low. According to the “Enemy Release Hypothesis” (ERH) invading hosts show reduced parasite burden by escaping the parasites in their native range when these are absent in the new range^10^. The ERH has empirical support in vertebrates including different bird species^10–18^. Generally, both parasite species richness and prevalence have been found to be lower in invasive than in native populations because specialist enemies (including their vectors) are often absent in the new region and host switching by specialist parasites of native species is rare^10,19^.

The “Evolution of Increased Competitive Ability Hypothesis” (EICA) posits that invasive plants that escape from parasites can reduce the investment in defence mechanisms and thereby allocate more energetic resources to reproduction and dispersal^20^. Lee and Klasing^9^ refined this hypothesis (revised-EICA) focusing on the vertebrate immune system. They suggested that successful vertebrate invaders are more likely to decrease investment in energetically costly immune defences and compensate with immunity that incurs less energetic costs. Thus, down regulated inflammatory responses which are costly might lead to an increase in less costly responses (e.g. antibody-mediated immunity)^9^. However, in contrast to the revised-EICA, Cornet et al.^21^ suggest that newly acquired local parasites might induce an equivalent immune response in invasive and native populations. Also, Brown and Shine^22^ suggest that trade-offs between different immune effectors are more important for invasive species than the general downregulation of costly traits. Moreover, Møller and Cassey^23^ propose that invasive bird species with strong immune response towards novel parasites are potentially more likely to become successful invaders. A better understanding of the role and complex interplay of parasites and immune effectors in the context of invasion is clearly necessary.

The main goals of this study were to compare parasite prevalences and immune effector levels between native and invasive Egyptian geese (*Alopochen aegyptiacus*) and shed light on their complex interplay in the frame of invasion. We tested (a) the prediction derived from the ERH that invasive populations are less infected than native populations and (b) the predicted differences in costly and less costly immune responses between native and invasive populations based on the revised-EICA hypothesis. Additionally, to test for potential differences in the strength of immune response between native and invasive birds, we did not restrict our analyses to investigating changes in mean levels of immune effectors. Instead, as an additional exploratory analysis, we studied how the variance in immune effectors differs between native and invasive populations, expecting changes in the variance of immune effectors in addition to changes in the mean levels. We chose a native and an invasive population of the Egyptian goose as one of the most successful invasive bird species in Europe. Its European population continues to increase^24,25^ and it has been listed as an invasive species in Germany according to “EU Regulation No 1143/2014 on the prevention and management of the introduction and spread of invasive alien species” and the BfN script 47 in 2017^26^.

## Material and methods

### Study species

The Egyptian goose is native to Africa and was introduced to Europe in the twentieth century^27^. Its native population is distributed on the sub-Saharan African continent and is one of the most common and wide spread African waterfowl species. Egg laying occurs throughout the whole year with a peak between late winter and early summer^28^.

The neozootic population invades Europe eastwards starting from the Netherlands, where they were introduced as ornamental species to parks^27^. It is now one of the most spreading neozootic bird species in Europe^24^. From the 1980s Egyptian geese also invade Germany where its population size increased rapidly^29,30^. The Egyptian goose is a resident (non-migratory / short distance migratory), monogamous, territorial bird species occurring as neozootic species in a variety of water habitats (e.g. streams, rivers, ponds, lakes,) in Europe^31^.

#### Sampling

Parasite prevalence and immunity of Egyptian geese from a native population in Namibia were investigated and compared to those of a currently spreading invasive population of the same species in Germany. In both regions, geese were sampled during ringing procedures (live trapped) or dissected after general pest control hunting (necropsy). Blood samples for immunological assays and serology exclusively originate from live trapped individuals whereas macro-parasite investigation was performed during necropsy. Micro-parasite investigation was performed in birds from both groups. Therefore, the resulting datasets are analysed separately (see method section: Statistical analysis) but a potential interplay between the different parasite prevalences and immune results is evaluated in the discussion.

#### Live trapping

Twenty-one Egyptian geese (9 male, 12 female) were live trapped in Namibia (22.35° S, 17.05° E) (native range) in February 2016. Additionally, data from a subset of 110 adult Egyptian geese from Germany (65 male, 45 female) investigated by Prüter et al.^32^ were included. German geese were sampled in the Rhine and Mosel areas (50.4° N, 7.6° E) (invasive range) in 2015 (*n* = 78) and 2016 (*n* = 32) in different months (supplementary data Table S1). Sex and reproductive status were recorded. Reproductive status was defined as breeding (e.g. guiding gosling, showing territorial behavior with a partner, having an egg-laying active cloaca) or non-breeding (e.g. not fulfilling criteria of breeding and/or being part of a non-family-flock). Numbers of breeding vs. non-breeding individuals can be found in Table S1. All Namibian birds were likely non-breeding individuals, which were sampled at a cattle feedlot were thousands of birds fly in to feed on the corn provided to the cattle. Blood was drawn from the vena *metatarsalia plantaris superficialis* using needles with a diameter of 0.06 mm for males and 0.04 mm for females. A fresh blood smear was prepared at capture and air dried. Blood samples were kept at 4–8 °C, centrifuged and sera were frozen in liquid nitrogen within eight hours after blood draw. Pharyngeal swabs were collected using sterile cotton swabs. Once field work finished, samples were transported to the Leibniz Institute for Zoo and Wildlife Research, Berlin, Germany and sera, blood clot and pharyngeal swabs were kept frozen at – 80 °C. Sampling in Germany was authorized by the Landesuntersuchungsamt Rheinland-Pfalz (G 15-20-005) and Landesamt für Natur, Umwelt und Verbraucherschutz Nordrhein-Westfalen (LANUV) (84-08.04.2015.A266). Permission to collect samples in Namibia was granted to GM and HK by the Ministry of Environment and Tourism (MET). Permission to export sample material from Namibia was granted by a MET export permit (No. 107513), and samples were transported to Germany in compliance with the Nagoya Protocol on Access and Benefit-sharing. All experimental procedures described in the materials and methods section were approved by the *Internal Committee for Ethics and Animal Welfare* of the Leibniz Institute for Zoo and Wildlife Research (permit #2014-11-03). All experiments were carried out in accordance with the approved guidelines of the Leibniz Institute for Zoo and Wildlife Research.

#### Necropsy

Additionally to live trapping, twenty-six free ranging Egyptian geese (17 male, 9 female) hunted during the autumn/winter season 2014/2015 and 2015/2016 in the North and West of Germany and twenty-seven Egyptian geese (11 male, 16 female), which were shot in February 2016 during regular pest control in Central Namibia were dissected. One of twenty-seven Namibian birds was live trapped and sampled before death and is thus included in both groups (live trapped and necropsy). Geese from Germany were kept frozen at – 20 °C after hunting until further analysis. Namibian geese were dissected immediately *post mortem*. During necropsy, ectoparasites, intestinal helminths and nasal leeches were collected. Additionally, pharyngeal swabs were taken for molecular analyses.

### Parasitological and microbiological analysis

Both macro-parasites (ectoparasites, nasal leeches (Euhirundidae), intestinal helminths) and selected micro-parasites (blood parasites (Haematozoa), bacteria, viruses) of Egyptian geese from the two populations were characterized for community composition and prevalence (methods see Table S2). Hereafter we use the term “parasites” combining macro- and micro-parasites and only explicitly distinguish between the type of parasites when differences can be expected and/or occur.

During necropsy, wing and breast feathers were macroscopically checked for the presence of ectoparasites. The upper beak was cut open and macroscopically investigated for the presence of nasal leeches. Intestinal helminths were extracted from the intestine of the birds and were determined to the family level based on morphology^33^. Additionally, blood smears of all live-trapped animals were investigated for the presence of blood parasites during white blood cell counts^34^.

To compare with previously determined bacterial prevalence of adult German Egyptian geese^32^ (Table 2), the Namibian birds were screened for *Mycoplasma* spp. and *Riemerella (R.) anatipestifer* using conventional 16S rRNA-based PCR assays as described by Prüter et al.^32^. To verify the specificity of the *Mycoplasma* PCR assay, products with a clear band were further investigated by sequence analysis, following the procedure described by Prüter et al.^32^. Only samples with a clear sequencing result were designated positive.

The seroprevalence of antibodies (Ab) against Influenza A virus (IAV), *Avian avulavirus 1* (AAvV-1) and *West Nile virus* (WNV) were determined^32^. For the detection of Abs against IAV, a commercial competitive enzyme linked immunosorbent assay (ELISA) was used following the manufacturer instructions (ID.vet, Grabels, France, Influenza A Antibody competition, FLUACA ver 0917DE). A commercial competitive ELISA for detection of Abs against AAvV-1 (Avian paramyxovirus 1; syn. Newcastle disease virus) was used according to the manufacturer protocol (ID.vet, Grabels, France, Newcastle Disease Competition, NDVC ver 0913 DE). Commercial competitive ELISA for Abs against Flaviviridae including WNV were applied following the manufacture protocol (ID.vet, West Nile Competition, WNC ver 1014-1P DE).

### Immunological assays

Several eco-immunological tests were used to quantify both the cellular and humoral parts of the acquired and innate immune responses of Egyptian geese^35^. Most of the methods are species-non-specific and have been used in a wide variety of free-living avian species, including different waterfowl^36–38^. We quantified the amounts of different humoral (natural antibodies, complement, lysozyme and haptoglobin) and cellular (monocytes, heterophils, eosinophils and basophils) effectors of innate immunity. For adaptive immunity we measured the total immunoglobulin Y (IgY) concentration and the number of lymphocytes^36^. Sample sizes (*n*) for each assay were dependent on the total amount of serum available from each individual and therefore differ among the tests (Table 1).

**Table 1:** Total sample sizes (total *n*), sample sizes grouped by sex (sex ratio (♂, ♀)) and year of sampling of blood and serum samples from Namibian (native) and German (invasive) Egyptian geese (*Alopochen aegyptiacus*) for each immunological effector grouped by the costs of immunity (low costs vs. high costs according to Klasing^39^ and Lee and Klasing^9^.

Immunological effectors	Invasive 2015 (Germany)		Invasive 2016 (Germany)		Native 2016 (Namibia)	
	Total n	Sex ratio (♂, ♀)	Total n	Sex ratio (♂, ♀)	Total n	Sex ratio (♂, ♀)
**Low cost**						
IgY	74	44, 30	26	16, 10	21	9, 12
Lysozyme	76	43, 33	30	18, 12	20	9, 11
Natural antibodies, complement	75	43, 32	24	16,8	21	9, 12
**High cost**						
Granulocytes (basophil, eosinophil, heterophil), Total leucocytes, Lymphocytes, Monocytes	77	45, 32	31	19, 12	21	9, 12
Haptoglobin	72	42, 30	23	15, 8	21	9, 12

#### Immunoglobulin Y

Total IgY, the avian equivalent to mammalian IgG, was measured using a sensitive ELISA with commercial anti-chicken antibodies^38,40^. Ninety six-well high-binding ELISA plates (82.1581.200, Sarstedt) were coated with 100 µl of diluted serum sample (2 samples per bird 1:16,000 diluted in carbonate–bicarbonate buffer) and incubated first for 1 h at 37 °C and then overnight at 4 °C. After incubation, the plates were washed with a 200 µl solution of phosphate buffer saline and PBS–Tween, before 100 µl of a solution of 1% gelatine in PBS–Tween was added. Plates were then incubated at 37 °C for 1 h, washed with PBS–Tween and 100 µl of polyclonal rabbit anti-chicken IgY conjugated with peroxidase (A-9046, Sigma) at 1:250 (v/v) was added. Following 2 h incubation at 37 °C, the plates were washed again with PBS–Tween three times. After washing, 100 µl of revealing solution [peroxide diluted 1:1000 in ABTS (2,20-azino-bis- (3-ethylbenzthiazoline-6-sulphonic acid))] was added, and the plates were incubated for 1 h at 37 °C. The final absorbance was measured at 405 nm using a photometric microplate reader (μQuant Microplate Spectrophotometer, Biotek) and subsequently defined as total serum IgY levels^41^.

#### Lysozyme

To measure lysozyme concentration in serum, we used the lysoplate assay^37^: 25 μl serum were inoculated in the test holes of a 1% Noble agar gel (A5431, Sigma) containing 50 mg/100 ml lyophilized *Micrococcus lysodeikticus* (M3770, Sigma), a bacteria which is particularly sensitive to lysozyme concentration. Crystalline hen egg white lysozyme (L6876, Sigma) (concentration: 1, 1.25, 2.5, 5, 6.25, 10, 12.5, 20 and 25 µg/ml) was used to prepare a standard curve for each plate. Plates were incubated at room temperature (25–27 °C) for 20 h. During this period, as a result of bacterial lysis, a clear zone developed in the area of the gel surrounding the sample inoculation site. The diameters of the cleared zones are proportional to the log of the lysozyme concentration. This area was measured three times digitally using the software ImageJ (version 1.48, http://imagej.nih.gov/ij/) and the mean was converted to a semi-logarithmic plot into hen egg lysozyme equivalents (HEL equivalents, expressed in μg/mL) according to the standard curve^42^.

#### Haemolysis–haemagglutination assay

The levels of the natural antibodies and complement were assessed by using a haemolysis–haemagglutination assay as described by^43^ adjusted to the limited volume of serum. After pipetting 15 μl of serum into the first two columns of a U-shaped 96-well microtitre plate, 15 μl sterile PBS were added to columns 2–12. The content of the second column wells was serially diluted (1:2) until the 11th column, resulting in a dilution series for each sample from 1/1 to 1/1024. The last column of the plate was used as negative controls, containing PBS only. Fifteen μl of 1% rabbit red blood cells (supplied as 50% whole blood, 50% Alsever’s solution, Envigo) suspension was added to all wells and incubated at 37 °C for 90 min. After incubation, in order to increase the visualisation of agglutination, the plates were tilted at a 45° angle at room temperature. Agglutination and lysis, which reflect the activity of the natural antibodies and the interaction between these antibodies and complement^43,44^, was recorded after 20 and 90 min, respectively. Haemagglutination is characterised by the appearance of clumped red blood cells, as a result of antibodies binding multiple antigens, while during haemolysis, the red blood cells are destroyed. Titres of the natural antibodies and complement were given as the log2 of the reciprocal of the highest dilution of serum showing positive haemagglutination or lysis, respectively^43,45^.

#### Haptoglobin

We measured haptoglobin concentrations with a commercial kit (TP801, Tri-Delta Diagnostics, Inc.) following the instructions of the manufacturer. Haptoglobin concentrations (mg/ml) in undiluted serum samples were calculated according to the standard curve on each plate^36^.

#### White blood cell counts

To count leucocytes, blood smears were prepared, air-dried and stained using Giemsa- and May-Grünwald staining. Smears were examined at 1,000 fold magnification with oil immersion and the relative number and types of leucocytes were assessed by counting 100 leucocytes. The number of white blood cells of different types was expressed per 10^4^ erythrocytes^45^.

### Statistical analyses

#### Parasite prevalence

To investigate potential differences in the prevalence of parasites between native and invasive Egyptian geese, we used Fisher’s exact tests because relatively low sample sizes of dissected animals did not allow to perform multivariate analysis.

#### Immunity

The means and variances of the different immune effectors were compared between the invasive and native Egyptian geese populations. To this end, we used an extension of commonly applied linear models. Linear models assume that the response variable *y* is a function of a linear combination of *n* predictor variables *x* with coefficients *c*_*0*_*,..,c*_*n*_ and an error *ε*:

1 $$y_{i} = c_{0} + c_{1} x_{1,i} + \cdots + c_{n} x_{n,i} + {\upvarepsilon }_{i} ,$$

where ε is the so-called residual variance which captures all the variation in the response variable that is not explained by the predictors. In linear models *ε* is assumed to be normally and independently distributed around zero. An additional usual assumption is that the variance of this distribution is a constant ${\upsigma }_{0}$, i.e.:

2 $${\upvarepsilon }_{i} = N\left( {0,{{ \sigma }}_{0} } \right),$$

which corresponds to the assumption of normally distributed residuals with homogeneous variance.

Thus, the estimated effects of the predictors *c*_*1*_*,..,c*_*n*_ only describe changes in the mean of the response variable, but not in the variance around that mean.

In our analysis, models were used in which the variance was allowed to be a linear function of some predictor variables *z* (which might be the same or different from the predictors *x* of the mean in Eq. 1), i.e.:

3 $${\upvarepsilon }_{i} = N\left( {0,{{ \sigma }}_{0} + {\upsigma }_{1} z_{1,i} + \cdots + {{ \sigma }}_{n} z_{n,i} } \right).$$

Thus, we were able to estimate simultaneously the effect and respective p-values of predictors *x* upon the variation in the mean of the response variable (Eq. 1) and also the effects and respective p-values of predictors *z* upon the residual variation around that mean (Eq. 3).

In order to appropriately capture all the potential variation in the response variables we used linear mixed-effects models (LMMs), which in addition to fixed effect predictors in Eq. (1) also included a random effect as a predictor of the mean. However, for enhanced clarity random effects were omitted in the equations above.

Different immune effectors were used as response variables (Table 1). As predictors for the mean sex (male vs. female), reproductive status (breeding vs. non-breeding) and invasion status (native vs. invasive) were included as fixed effects and month of sampling as a random effect. In this way, we control for potential confounding effects of breeding status between the two populations. As predictors for the variance, we included invasion status (native vs. invasive), sex and reproductive status, which allowed us to test our prediction that the variance in immune effects is higher among invasive individuals compared to native individuals.

Some of the immune effectors were transformed (see tables supplementary data S3, S4, S5) to ensure normality of residuals. For haptoglobin we were not able to perform a transformation that ensured normality, because of the high proportion of values below the detection threshold. To account for this, we performed a generalized linear mixed model (GLMM) with a binominal error distribution and with a binary response variable (haptoglobin being either above or below the detection threshold). In this model, it was necessary to constrain the error variance to a fixed value. Thus, for haptoglobin we were only able to test for a change in mean but not for a change in variance. In addition to analysing total leucocytes, different leucocyte subtypes were analysed separately.

The LMMs and GLMMs were implemented using the R package *glmmTMB* version 0.2.0^46^. To test for differences in residual variance we used the option *disformula* in the function *glmmTMB.* Potential collinearity of predictors was tested by calculating variance inflation factors using the R package *car* version 2.1-6^47^*.* All statistical analyses were performed using R version 3.3.2^48^.

## Results

### Parasite prevalences

Blood parasites and all groups of macro-parasites (ectoparasites, nasal leeches, intestinal cestodes and trematodes) were found at lower prevalence in the invasive than in the native population of Egyptian geese with the exception of intestinal nematodes. However, none of the observed trends reached statistical significance (Table 2). The bacterium *R. anatipestifer,* was found in 67.0% of adult invasive Egyptian geese from Germany^32^ but was not detected in native geese from Namibia. This difference was statistically significant (Table 2). *Mycoplasma* spp*.,* which was not detected in the German geese^32^, was detected in two individuals from Namibia. However, the result was not statistically significant (Table 2). Seroprevalence of selected viruses (IAV, AAvV and WNV) were lower in geese from the invasive population but the difference was only statistically significant for antibodies against IAV (Table 2).

**Table 2:** Results of the parasite screening and serology of Egyptian geese from Namibia and Germany (adult geese from Prüter et al.^32^).

	Namibia			Germany			Trend	Fisher test
	*n*	Infected	Prevalence	*n*	Infected	Prevalence		*p*
**Parasitology**								
Group								
Ectoparasites	27	11	40.74	26	3	11.54	↓	0.08
Euhirundidae	27	1	3.85	26	0	0	↓	1
Intestinal helminths	27	6	22.2	26	4	15.38	↓	0.74
Cestoda	27	2	7.4	26	1	3.8	↓	1
Nematoda	27	0	0	26	1	3.8	↑	1
Trematoda	27	4	14.8	26	1	3.8	↓	0.36
Haematozoa	21	1	4.76	110	0	0	↓	0.17
**Bacteria**								
Target genes								
*Riemerella anatipestifer* 16S rRNA gene	47	0	0	94	63	67.02	**↑**	** < 0.001**
*Mycoplasma* spp. 16S rRNA gene	47	2	4.44	94	0	0	↓	0.12
**Serology**								
Antigen								
IAV	21	9	42.86	105	9	8.57	**↓**	**0.003**
AAvV-1	20	2	10	102	4	3.92	↓	0.27
WNV	13	1	7.69	56	0	0	↓	0.2

Total sample sizes (*n*), number of infected individuals (Infected) and prevalences (%) of macro-parasites, bacteria and seroprevalences against selected viruses in the native Namibian and invasive German population of Egyptian geese (*Alopochen aegyptiacus*); Trend: ↓ higher prevalence in the native than in the invasive population; ↑ higher prevalence in the invasive than in the native population; Outcome of Fisher’s exact test comparing prevalences of the two groups (*p*-value < 0.05 is defined as significant and indicated in bold.

*IAV* Influenza A virus, *AAvV-1* *Avian avulavirus 1*, *WNV* *West Nile virus*.

### Immunity

#### Differences in mean immune function measures

**Figure 1:**
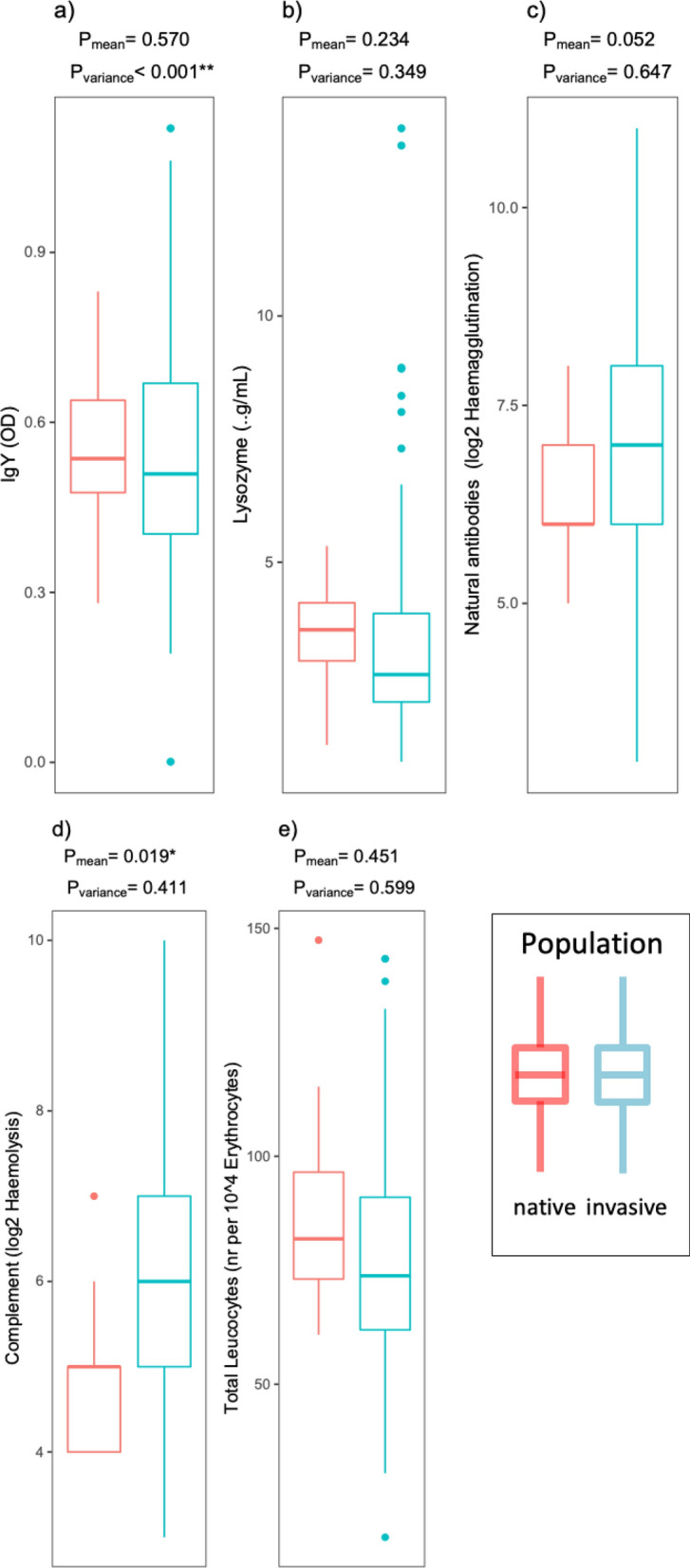
Differences in distributions of low cost (**a**–**d**) and high cost (**e**) immune measures between native and invasive Egyptian geese are shown; red = native; blue = invasive; P = *p*-values for the effects of population (native vs. invasive) on the mean (P_mean_) and variance (P_variance)_ of the respective immune measure from the GLMMs (see Tables S3, S4). For sample sizes of each immune measure for the two populations see Table 1.

**Figure 2:**
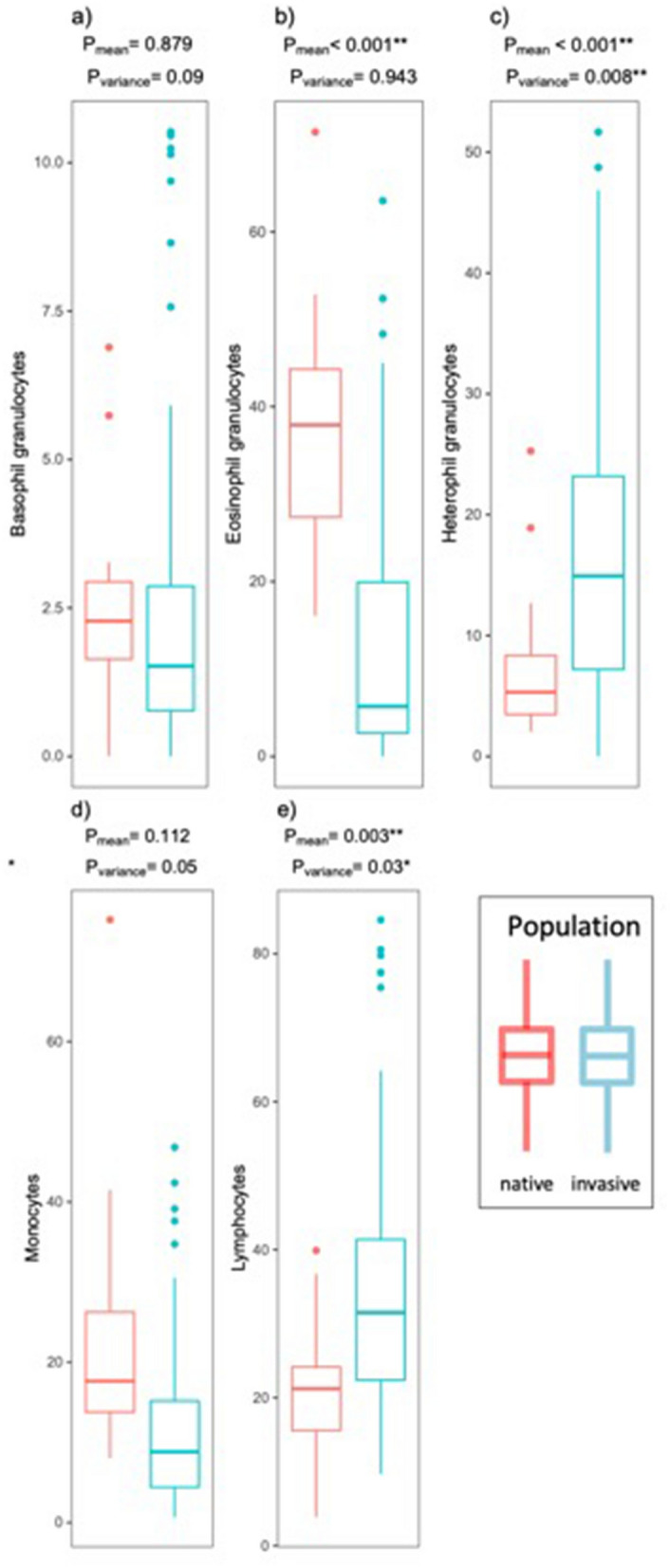
Differences in distributions of the differential white blood cells (nr per 10^4^ erythrocytes) (**a**–**e**) between native and invasive Egyptian geese are shown; red = native; blue = invasive; P = *p*-values for the effects of population (native vs. invasive) on the mean (P_mean_) and variance (P_variance)_ of the respective immune measure from the GLMMs (see Table S5). For sample sizes of each immune measure for the two populations see Table 1.

**Figure 3:**
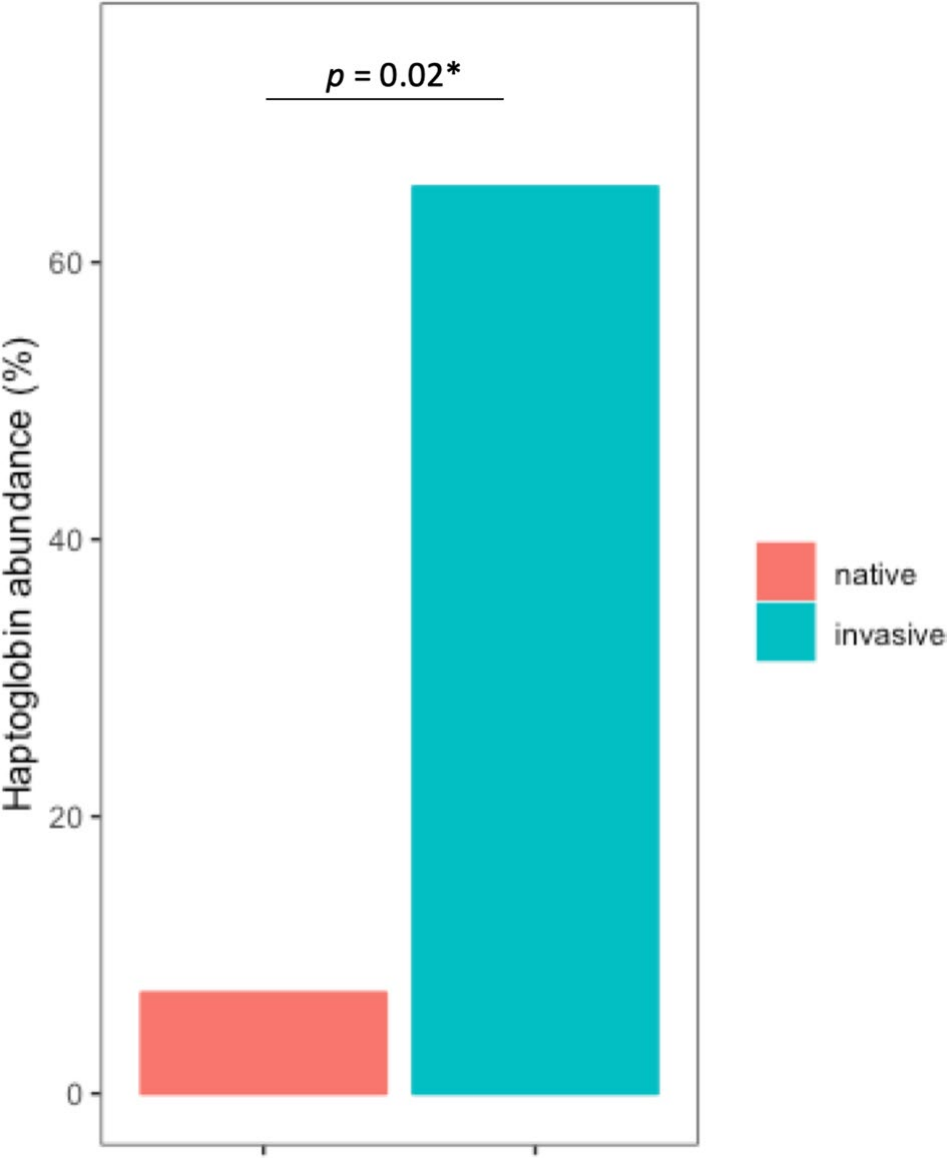
A barplot for the differences in haptoglobin abundance (percent of individuals with haptoglobin concentrations above the detection threshold) between native and invasive Egyptian geese is shown; red = native, blue = invasive; *p*-value for the effects of population (native vs. invasive) on the mean haptoglobin from the binomial distribution model from the GLMMs (see Table S4). (It was not possible to fit a Gaussian model for haptoglobin concentration. Thus, the assessment of the difference in variance was not possible).

Of the four assays measuring ‘low cost’ immune effectors, mean haemolysis was significantly higher in the invasive population. Mean IgY, mean lysozyme and mean haemagglutination did not significantly differ between the two study populations (Fig. 1). Among the ‘high cost’ immune effectors, the mean number of leucocytes was not significantly different between the two study populations (Fig. 1). Detailed analyses of the means of differential white blood cells (Fig. 2) demonstrated significantly higher mean numbers of heterophils and lymphocytes in the invasive population, whereas mean eosinophil concentration was significantly lower. No statistically significant differences in mean monocyte and basophil numbers were observed between the two groups. In contrast, haptoglobin abundance was significantly higher in the invasive (65%) than in the native (7.3%) population (Fig. 3).

Males showed significantly higher mean levels of IgY, haptoglobin abundance, total leucocytes and lymphocytes than females. Mean lymphocytes were significantly higher in birds that were in breeding status than in non-breeding individuals. No statistically significant effects on the other means of measured immune effectors were associated with sex or reproductive status (supplementary data Tables S3, S4 and S5).

#### Variance of immune effector measures

Among the ‘low cost’ immune effectors the variance of IgY was significantly higher in the invasive population. However, the variance of lysozyme, haemagglutination, haemolysis did not differ significantly between the two groups (Fig. 1). For the ‘high cost’ immune effectors, the variance of total leucocytes did not differ significantly between the two groups (Fig. 1). The variance in the concentration of lymphocytes and heterophils was significantly higher in the invasive geese. Variance in basophil, eosinophil and monocyte counts was not significantly different between the groups (Fig. 2).

Males showed signifficantly higher variance of haemagglutinaion than females. The reproductive status of the birds significantly influenced the variance of three ‘low cost’ immune effectors. The variance of IgY was signifficantly higher in breeding than in non-breeding birds whereas the variance of lysozyme and haemagglutination was signifficantly lower in breeding birds than in non-breeding individuals (supplementary data Tables S3, S4 and S5).

## Discussion

### The results are not consistent with the revised-EICA

Four out of eleven immune effectors had higher mean values in invasive population when compared to their native conspecifics. Three of these immune effectors are considered energetically costly^9^, which is inconsistent with the predictions of the revised-EICA hypothesis which predicts a shift to low cost immune effectors. In line with the ERH, all but one of the parasites examined decreased in prevalence when comparing native and invasive populations of Egyptian geese, although this effect was not statistically significant. Only the bacterium *R. anatipestifer* was absent in the native population while the invasive population showed a 67% prevalence, which could indicate that the invading geese have encountered a novel pathogen during the invasion process^32^.

Most studies of invasive vertebrate hosts and their pathogens focus on helminths, which elicit a host Th2 response (e.g. promotes antibody production) and increase eosinophil numbers, and show reduced helminth infection and reduced eosinophil numbers in invasive populations compared to native^14,18,49,50^. Previous support for the revised-EICA related predictions have focused on macro-parasites, which might be biased toward the pathogens and immune effectors investigated. We observed low mean eosinophil numbers in invasive geese indicating a lower impact of macro-parasites on the immune system than in the native population (consistent with the ERH and the revised-EICA).

*R. anatipestifer* is a bacterium of relevance for domestic ducks and geese, leading to severe clinical symptoms^51,52^. The lack of clinical symptoms in the invasive Egyptian geese infected with *R. anatipestifer* may indicate that they are more resistant or tolerant than expected^21,53^ or this waterfowl may carry *R. anatipestifer* as a commensal bacterium^32,54^. Overall, immune defences effective against micro-parasites, especially bacteria (heterophils, lymphocytes, haemolysis) were found to be higher on average in the invasive than in the native goose populations. Haptoglobin in particular, which is an energetically costly acute phase protein with bacteriostatic function^36^, was significantly more abundant in the invasive than in the native population of Egyptian geese which is inconsistent with the EICA-related predictions.

Studying a variety of micro- and macro-parasites and high numbers of different immune effector levels—as done in this study- indicate that presence or absence of support for existing hypotheses likely depends on the parasite and immune response investigated. This demonstrates that existing hypotheses predicting immune effector levels and parasite prevalence in invasive populations are likely too simplistic considering the loss and gain of parasites as well as the complexity of immune responses.

### Immune effector variance

The variance for three out of ten immune effectors in the invasive population of Egyptian geese was higher than in the native population. In our opinion, existing hypotheses, such as the revised EICA, insufficiently cover the complex interplay of loss and gain of parasites in invasive populations. The higher variance of some immune effectors in the invasive population might indicate higher immune plasticity on the individual level (as measured here) which could reflect higher variation in immune reaction on the population level. Higher variation in immune reaction might enable invaders to efficiently defend against novel pathogens while simultaneously reducing the overall costs of immunity. The complexity of the immune system and the specificity of different immune effectors to specific parasites may be more important during invasion than previously acknowledged^55^. Furthermore, White and Perkins^56^ suggested that higher plasticity of individual immune response in a founder population, the more likely the species will successfully invade. Ghalambor et al.^57^ hypothesized that the period of persistence of invasive species (period after introduction and before rapid population growth starts) might be dependent on phenotypic plasticity. Changes in immune investment and variation of immune reactions in the different stages of invasion are likely (for the different phases of invasion see Duncan et al.^5^), although the underlying cause of a variation of immune reactions might differ among invasive populations (e.g. genetically heterogeneous populations, diversity of outside stimuli). Variation of immunity, as opposed to generally reduced investment in immune function, might provide invading species with the necessary flexibility to colonize novel environments. Specifically, flexibility of immune reactions could enable invaders to balance reduced immune investment against their original parasites with increased defence against novel parasites. Individuals not confronted with novel parasites could reduce their immune investment (in line with the revised-EICA hypothesis), whereas infected individuals could selectively increase the appropriate immune defense. Here, a significantly higher mean value of heterophils, as effectors against bacterial infections, with evidence for higher variance in the invasive population indicate that pathogenic bacteria (as the here selected *R. anatipestifer*) might be particularly important to the invasive population.

**Figure 4:**
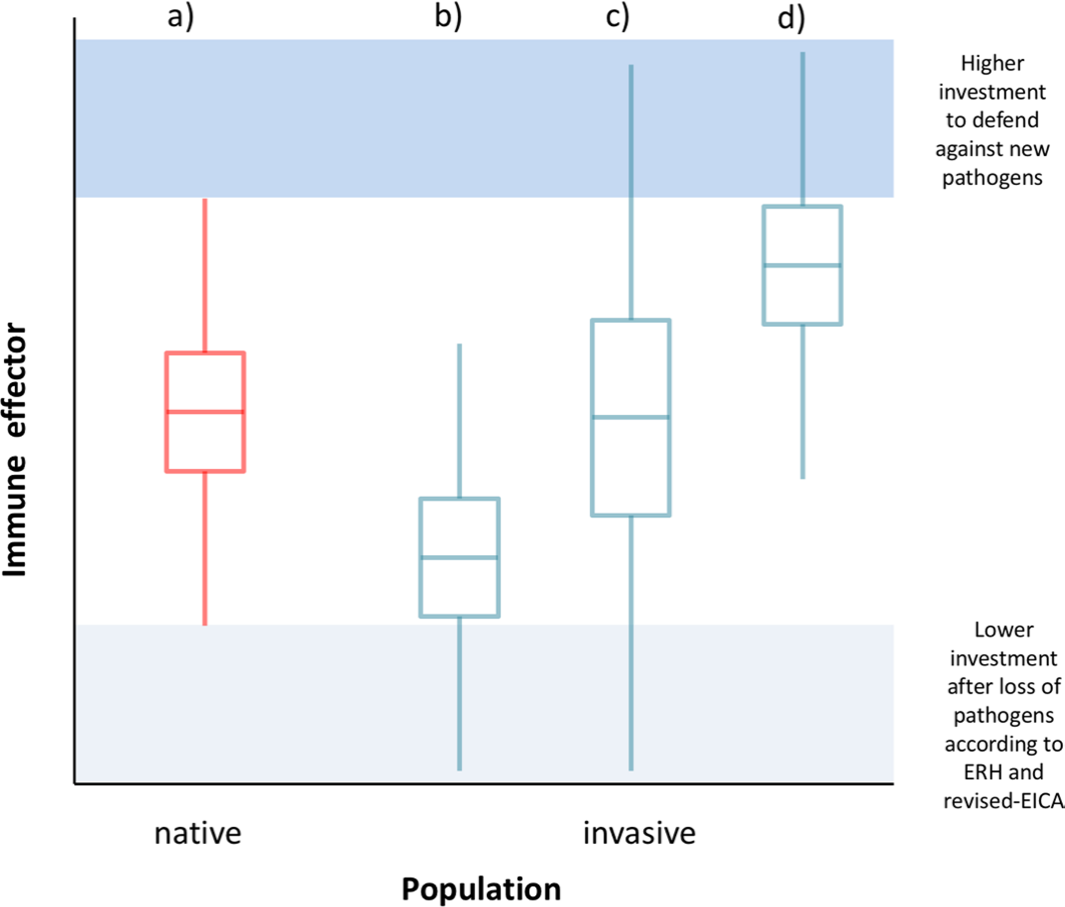
Predictive changes of the means and variances of immune effectors between native (**a**) and invasive (**b**–**d**) populations; (**b**) decrease in mean according to the “Enemy Release Hypothesis” (ERH) and “revised-Evolution of Increased Competitive Ability Hypothesis” (revised-EICA), (**c**) increase in variance if the effect of enemy release allows for decreasing investment in immunity but defence against new parasites must increase as new parasites infect the hosts, (**d**) the effect of increased investment to defend against new parasites is stronger than the enemy release effect.

Depending on the immune effector and its role in the immune response against parasites and the stage of the invasion process we propose three scenarios consistent with our results (Fig. 4b–d). If the enemy release effect is dominant (e.g. early stages of invasion; invasion front), immune investment would be down-regulated compared to native populations (Fig. 4a) as suggested by the revised-EICA hypothesis (Fig. 4b). In this case, low parasite prevalence and reduced overall costs of immunity would be expected. During range expansion, members of invasive populations would likely increasingly face novel parasites. Invasive animals must have effective defences against diverse newly encountered parasites. Heterogeneous prevalence for parasite species would be expected with greater heterogeneity expected during the early stages of invasion. Some invading individuals would need to be able to defend themselves against the new parasites requiring higher immune investment than observed in native individuals^23^. Individuals of the invading population not infected may invest less in immunity compared to their native relatives. Variation in immune investment would be expected to be prominent during early stages of invasion and accordingly we would expect an increased variance in immune measures (Fig. 4c). In later phases, prevalence of parasites might increase further and thus, a need to defend against new parasites (Fig. 4d).

### Eco-immunology of Egyptian geese

The Egyptian goose (*Alopochen aegyptiacus*) is a successful invasive bird species having and continuing to spread throughout Western Europe since the mid-twentieth century^25^. The impact of newly acquired pathogens on this population is most likely still ongoing and potentially increasing (middle phase of invasion, Fig. 4c). That the invasion is still on-going likely explains why increased immune effector variance is prominent. Our results indicate that among-individual variation in immunity is on average higher in invasive compared to native Egyptian geese but that these effects might be parasite dependent and therefore the effects differ among immune effectors.

In contrast to parasite infection, the higher bactericidal investment but less variance in bacterial related immunity, might indicate that population growth rates will slow in the future, as immune investment increases and consequently energy reallocates away from reproduction. After the invasion has progressed further or has been completed, the variance of all effectors is expected to decrease representing adaptation to the new environment (Fig. 4d).

When interpreting our results, it is important to consider the limitations of our study. First, sample sizes, especially of dissected animals, are low and immune measures and macro-parasite data originate from different individuals and thus preclude a direct analysis of the interplay between immune marker level and infection status. Additionally, low sample sizes and sample size differences between the populations may impact the statistical power of the analysis. For future studies, we recommend avoiding this bias to allow statistical testing for the interplay of specific immune markers and the different parasitic infections, which could clarify the role of different parasites the related immune responses within the invasion process. Increasing the overall sample size and aligning the sample sizes of the compared populations could reveal more statistically significant differences in the variance and mean of immune effectors between native and invasive populations. Additionally, bigger sample sizes would allow to test for the interaction between sex and reproductive status in the context of immunity and invasion.

Second, the degree of genetic relatedness between the investigated invasive and native population is unknown. Invasive populations can have higher or lower genetic diversity than native conspecifics^58,59^. Variance in immunity might either compensate for potentially lower genotypic variation of invasive populations or reflect higher genetic diversity. Here we cannot distinguish if the observed variation is caused by one or the other. Studies of the genotypic diversity of the immune system (e.g. MHC and other immune genes)^60^ of Egyptian geese from both populations could help to clarify the underlying mechanisms of the higher variance in immune effectors between the two populations in this study.

Third, local conditions (e.g. climate, habitats, community structures) of the two populations substantially differ. This differences in local conditions, which is a general aspect in invasion biology, clearly limits the interpretation of our results. Future studies of Egyptian geese populations (and those of other invasive species) should aim to increase the number of populations tested on a global scale to allow to control for local condition variables and their interplay with health indices of native and invasive populations^61^.

## Conclusions

The interplay of parasites and host immunity in the frame of invasion biology is complex and still not clarified in detail. Its role likely depends on the characteristics of the parasites and the resulting costs of host’s immune investment^9^. However, our study emphasizes that for unravelling the complexity of this interaction it could be very useful to measure both, mean and variance of different immune effectors in the light of parasitic infections. This could help to better understand contradictory findings in eco-immunological studies.

Contradictory effects of invasion on immune function have been reported for amphibians. Immune defences are weaker in invasive cane toads (*Rhinella marina*) that invade over long distances indicating a trade-off between dispersal and immune investment^22^. Cane toads on the invasion-front were found to have higher bactericidal and phagocytic activity than in more established populations^62^ consistent with our observations in Egyptian geese. However, the overall immune investment in cane toads at the invasion front was lower compared to established cane toad populations^63^. In contrast, Cuban tree frogs (*Osteopilus septentrionalis*) on the invasion front exhibited reduced bactericidal ability compared to established frogs^64^. Studying flexibility of the immune system during range expansion of these amphibians might help to explain the contradictory findings. We expect that higher immune reaction flexibility would benefit individuals of invasive populations (1) in the early stages of invasion by reducing investment in the immune system and (2) in later stages of invasion by providing effective defence against novel pathogens.

Intraspecific comparisons^21,65^ will be essential to determine if flexible immune effector response is a general principle during biological invasions^36^ allowing to allocate resources away from immunity whenever the parasitic burden is low. Common garden experiments combined with immune challenges or experimental infections would help to clarify the susceptibility to infection during range expansion of invasive species. Re-analysing data from previous studies based on the revised-EICA by investigating changes in the variance of immune effectors may also be a viable approach to determining if the results from Egyptian geese are relevant to other species. These approaches could help identify the drivers of successful invasion for neozootic species. Moreover, the interplay of local conditions and the strength of immune responses (variance) could add a new dimension to the field of invasion biology with predictive power regarding population dynamics of invasive populations in general.

## Supplementary Information

Supplementary Tables

## Data Availability

Data are available on “Dryad” public repository (10.5061/dryad.t4b8gtj0s).

## References

[1] Lövei GL (1997). Global change through invasion: biodiversity. Nature.

[2] McGeoch MA (2010). Global indicators of biological invasion: species numbers, biodiversity impact and policy responses: Invasive alien species indicator: 2010 Biodiversity Target. Divers. Distrib..

[3] Strauss A, White A, Boots M (2012). Invading with biological weapons: the importance of disease-mediated invasions. Funct. Ecol..

[4] Pyšek P, Richardson DM (2010). Invasive species, environmental change and management, and health. Annu. Rev. Environ. Resour..

[5] Duncan RP, Blackburn TM, Sol D (2003). The ecology of bird introductions. Annu. Rev. Ecol. Evol. Syst..

[6] Shea K, Chesson P (2002). Community ecology theory as a framework for biological invasions. Trends Ecol. Evol..

[7] Sheldon BC, Verhulst S (1996). Ecological immunology: costly parasite defences and trade-offs in evolutionary ecology. Trends Ecol. Evol..

[8] van der Most PJ, de Jong B, Parmentier HK, Verhulst S (2011). Trade-off between growth and immune function: a meta-analysis of selection experiments: trade-off between growth and immune function. Funct. Ecol..

[9] Lee KA, Klasing KC (2004). A role for immunology in invasion biology. Trends Ecol. Evol..

[10] Torchin ME, Lafferty KD, Dobson AP, McKenzie VJ, Kuris AM (2003). Introduced species and their missing parasites. Nature.

[11] Shwartz A, Strubbe D, Butler CJ, Matthysen E, Kark S (2009). The effect of enemy-release and climate conditions on invasive birds: a regional test using the rose-ringed parakeet (*Psittacula krameri*) as a case study. Divers. Distrib..

[12] Lima, M., Simpson, L., Fecchio, A. & Kyaw, C. Low prevalence of haemosporidian parasites in the introduced house sparrow (*Passer domesticus*) in Brazil. *Acta Parasitologica* **55**, (2010).

[13] Marzal A (2011). Diversity, loss, and gain of Malaria parasites in a globally invasive bird. PLoS ONE.

[14] Valente R (2014). Helminth parasites of the European starling (*Sturnus vulgaris*) (Aves, Sturnidae), an invasive bird in Argentina. Parasitol. Res..

[15] Clark NJ, Olsson-Pons S, Ishtiaq F, Clegg SM (2015). Specialist enemies, generalist weapons and the potential spread of exotic pathogens: malaria parasites in a highly invasive bird. Int. J. Parasitol..

[16] Ellis VA (2017). Prevalence of avian haemosporidian parasites is positively related to the abundance of host species at multiple sites within a region. Parasitol. Res..

[17] Keogh CL, Miura O, Nishimura T, Byers JE (2017). The double edge to parasite escape: invasive host is less infected but more infectable. Ecology.

[18] Diagne C (2016). Parasites and invasions: changes in gastrointestinal helminth assemblages in invasive and native rodents in Senegal. Int. J. Parasitol..

[19] Keane R, Crawley MJ (2002). Exotic plant invasions and the enemy release hypothesis. Trends Ecol. Evol..

[20] Blossey B, Notzold R (1995). Evolution of increased competitive ability in invasive nonindigenous plants: a hypothesis. J. Ecol..

[21] Cornet S, Brouat C, Diagne C, Charbonnel N (2016). Eco-immunology and bioinvasion: revisiting the evolution of increased competitive ability hypotheses. Evol. Appl..

[22] Brown GP, Shine R (2014). Immune response varies with rate of dispersal in invasive cane toads (*Rhinella marina*). PLoS ONE.

[23] Møller AP, Cassey P (2004). On the relationship between T-cell mediated immunity in bird species and the establishment success of introduced populations. J. Anim. Ecol..

[24] Bauer H-G, Woog F (2008). Nichtheimische Vogelarten (Neozoen) in Deutschland, Teil I: Auftreten, Bestände und Status—non-native and naturalized bird species (neozoa) in Germany, part I: occurrence, population size and status. Vogelwarte.

[25] Gyimesi, A. & Lensink, R. Risk analysis of the Egyptian Goose in the Netherlands. in *Bureau Waardenburg BV* (2010).

[26] Nehring, S. & Skowronek, S. Die invasiven gebietsfremden Arten der Unionsliste der Verordnung (EU) Nr. 1143/2014. Erste Fortschreibung 2017. in (Bundesamt für Naturschutz, 2017).

[27] Gyimesi, A. & Lensink, R. Egyptian Goose Alopochen aegyptiaca: an introduced species spreading in and from the Netherlands. *Wildfowl* 128–145 (2012).

[28] Brown, L. H., Urban, E. K. & Newman, K. *The birds of Africa*. vol. Volume 1 (New York: Academic Press., 1982).

[29] Arnold, J. M., Greiser, G., Kampmann, S. & Martin, I. Status und Entwicklung ausgewählter Wildtierarten in Deutschland. Jahresbericht 2013. *Wildtier-Informationssystem der Länder Deutschlands (WILD). Deutscher Jagdverband; Berlin* (2013).

[30] *Atlas Deutscher Brutvogelarten—Atlas of German breeding birds*. (Dachverband Deutscher Avifaunisten, 2015).

[31] Dietzen, C., Dolich, T., Grunwald, T., Keller, P. & Kunz, A. Die Vogelwelt von Rheinland-Pfalz. Band 2 Entenvögel bis Storchenvögel (Anseriformes-Ciconiformes). in *Gesellschaft für Ornithologie Rheinland-Pfalz* (2015).

[32] Prüter H (2018). Sane and sound: a serologic and molecular survey for selected infectious agents in neozootic Egyptian geese (Alopochen aegyptiacus) in Germany. Eur. J. Wildl. Res..

[33] *Lehrbuch der Parasitologie für die Tiermedizin: 112 Tabellen*. (Enke, 2008).

[34] Doster, G. L. & Goater, C. P. Collection and quantification of avian helminths and protozoa. in *Host-parasite evolution–general principles and avian models* 396±418 (Oxford University Press, 1997).

[35] Demas GE, Zysling DA, Beechler BR, Muehlenbein MP, French SS (2011). Beyond phytohaemagglutinin: assessing vertebrate immune function across ecological contexts: assessing vertebrate immune function across ecological contexts. J. Anim. Ecol..

[36] Matson KD, Cohen AA, Klasing KC, Ricklefs RE, Scheuerlein A (2006). No simple answers for ecological immunology: relationships among immune indices at the individual level break down at the species level in waterfowl. Proc. R. Soc. B Biol. Sci..

[37] Giraudeau M (2010). Effect of restricted preen-gland access on maternal self maintenance and reproductive investment in Mallards. PLoS ONE.

[38] Bourgeon S, Kauffmann M, Geiger S, Raclot T, Robin J-P (2010). Relationships between metabolic status, corticosterone secretion and maintenance of innate and adaptive humoral immunities in fasted re-fed mallards. J. Exp. Biol..

[39] Klasing KC (2004). The costs of immunity. Acta Zool. Sin..

[40] Martinez J, Tomas G, Merino S, Arriero E, Moreno J (2003). Detection of serum immunoglobulins in wild birds by direct ELISA: a methodological study to validate the technique in different species using antichicken antibodies. Funct. Ecol..

[41] Bourgeon S, Raclot T (2006). Corticosterone selectively decreases humoral immunity in female eiders during incubation. J. Exp. Biol..

[42] Rowe M, Czirják GÁ, Lifjeld JT, Giraudeau M (2013). Lysozyme-associated bactericidal activity in the ejaculate of a wild passerine: Lysozyme in the ejaculate of a wild bird. Biol. J. Lin. Soc..

[43] Matson KD, Ricklefs RE, Klasing KC (2005). A hemolysis–hemagglutination assay for characterizing constitutive innate humoral immunity in wild and domestic birds. Dev. Comp. Immunol..

[44] Pap PL, Czirják GÁ, Vágási CI, Barta Z, Hasselquist D (2010). Sexual dimorphism in immune function changes during the annual cycle in house sparrows. Naturwissenschaften.

[45] Pap PL (2015). Physiological pace of life: the link between constitutive immunity, developmental period, and metabolic rate in European birds. Oecologia.

[46] Brooks ME (2017). Modeling Zero-Inflated Count Data With glmmTMB..

[47] Fox, J. & Weisberg, S. An R companion to applied regression. *CA:SAGE Publications Inc.* 2nd ed. Thousand Oaks, (2011).

[48] R Core Team. *R: A language and environment for statistical computing*. (R Foundation for Statistical Computing, 2016).

[49] Al-Sabi MNS, Chriél M, Jensen TH, Enemark HL (2013). Endoparasites of the raccoon dog (*Nyctereutes procyonoides*) and the red fox (*Vulpes vulpes*) in Denmark 2009–2012: a comparative study. Int. J. Parasitol. Parasites Wildl..

[50] Romeo C (2014). Macroparasite fauna of alien grey squirrels (*Sciurus carolinensis*): composition, variability and implications for native species. PLoS ONE.

[51] Hubálek Z (2004). An annotated checklist of pathogenic microorganisms associated with migratory birds. J. Wildl. Dis..

[52] Hinz K, Ryll M, Köhler B, Glünder G (1998). Phenotypic characteristics of *Riemerella anatipestifer* and similar micro-organisms from various hosts. Avian Pathol..

[53] Raberg L, Graham AL, Read AF (2009). Decomposing health: tolerance and resistance to parasites in animals. Philos. Trans. R. Soc. B: Biol. Sci..

[54] Ryll, M. *et al.* Studies on the prevalence of Riemerella anatipestifer in the upper respiratory tract of clinically healthy ducklings and characterization of untypable strains. *J. Vet. Med. B Infect. Dis. Vet. Public Health* **48**, 537–546 (2001). 10.1046/j.1439-0450.2001.00471.x 11666036

[55] Morand S (2015). Global parasite and *Rattus* rodent invasions: the consequences for rodent-borne diseases. Integr. Zool..

[56] White TA, Perkins SE (2012). The ecoimmunology of invasive species. Funct. Ecol..

[57] Ghalambor CK, Mc Kay JK, Carroll SP, Reznick DN (2007). Adaptive versus non-adaptive phenotypic plasticity and the potential for contemporary adaptation in new environments. Funct. Ecol..

[58] Roman J, Darling J (2007). Paradox lost: genetic diversity and the success of aquatic invasions. Trends Ecol. Evol..

[59] Edelaar P (2015). Shared genetic diversity across the global invasive range of the monk parakeet suggests a common restricted geographic origin and the possibility of convergent selection. Mol. Ecol..

[60] Acevedo-Whitehouse K, Cunningham A (2006). Is MHC enough for understanding wildlife immunogenetics?. Trends Ecol. Evol..

[61] Becker DJ (2020). Macroimmunology: The drivers and consequences of spatial patterns in wildlife immune defence. J. Anim. Ecol..

[62] Brown GP, Phillips BL, Dubey S, Shine R (2015). Invader immunology: invasion history alters immune system function in cane toads (*Rhinella marina*) in tropical Australia. Ecol. Lett..

[63] Llewellyn D, Thompson MB, Brown GP, Phillips BL, Shine R (2012). Reduced investment in immune function in invasion-front populations of the cane toad (*Rhinella marina*) in Australia. Biol. Invasions.

[64] Goetz SM, Romagosa CM, Appel AG, Guyer C, Mendonça MT (2018). Reduced innate immunity of Cuban treefrogs at leading edge of range expansion. J. Exp. Zool. Part A Ecol. Integr. Physiol..

[65] Roy HE, Lawson Handley L-J (2012). Networking: a community approach to invaders and their parasites. Funct. Ecol..

