# Supplementary material for: Increased immune marker variance in a population of invasive birds
Source: Sci Rep. 2020 Dec 10;10:21764. doi: 10.1038/s41598-020-78427-7 (PMC7729907; doi:10.1038/s41598-020-78427-7)
Supplement: Supplementary file 1 — Supplementary Tables [file 41598_2020_78427_MOESM1_ESM.pdf]

Increased immune marker variance in a population of invasive birds

Prüter, Hanna[1]; Franz, Mathias[1]; Twietmeyer, Sönke[2]; Böhm, Niklas[3]; Middendorff, Gudrun[4]; Portas, Ruben[5]; Melzheimer, Jörg[5]; Kolberg, Holger[6]; von Samson-Himmelstjerna, Georg[7]; Greenwood, Alex D.[1,8]; Lüschow, Dörte[9]; Mühldorfer, Kristin[1]; Czirják, Gábor Árpád[1]

[1] Leibniz Institute for Zoo and Wildlife Research, Department of Wildlife Diseases, Alfred-Kowalke-Straße 17, 10315 Berlin, Germany
[2] Department of Research and Documentation, Eifel National Park, Urftseestraße 43, 53937 Schleiden-Gemünd, Germany
[3] FÖA Landschaftsplanung GmbH, Auf der Redoute 12, 54296 Trier, Germany
[4] Namibia Bird Club, Windhoek, Namibia
[5] Leibniz Institute for Zoo and Wildlife Research, Department of Evolutionary Ecology, Alfred-Kowalke-Straße 17, 10315 Berlin, Germany
[6] Ministry of Environment and Tourism, Private Bag, 13306 Windhoek, Namibia
[7] Freie Universität Berlin, Institute for Parasitology and Tropical Veterinary Medicine, Robert-von-Ostertag-Str. 7-13, 14163, Berlin, Germany
[8] Freie Universität Berlin, Department of Veterinary Medicine, 14163 Berlin, Germany
[9] Freie Universität Berlin, Institute of Poultry Diseases, Königsweg 63, 14163, Berlin, Germany

Supplementary data

Table S1:

Month of sampling	Status	Origin	non-breeding		breeding	
			male	female	male	female
**2015**						
April	invasive	Germany	1	0	7	4
June	invasive	Germany	1	1	17	12
July	invasive	Germany	3	0	11	11
October	invasive	Germany	2	3	2	2
November	invasive	Germany	1	0	0	0
**2016**						
February	native	Namibia	9	12	0	0
June	invasive	Germany	1	0	12	9
July	invasive	Germany	2	0	5	3

Table S2: Summary output of the GLMMs to test for differences in the mean (in the conditional model) and variance (in the dispersion model) of "low cost" immune effectors.

Effect on the mean						Effect on the variance				
**Response**	**Predictor (fixed effects)**	**Estimate**	**Std. Error**	**z**	**p**		**Estimate**	**Std. Error**	**z**	**p**
Igy	Intercept	0.75685	0.02033	37.24	<0.001 ***	Intercept	-3.9567	0.2212	-17.888	<0.001 ***
	status	0.03471	0.06113	0.57	0.570	status	-1.8013	0.4984	-3.614	<0.001 ***
	sex	-0.05434	0.02341	-2.32	0.020 *	sex	-0.1757	0.2741	-0.641	0.521
	reproduction	-0.03193	0.05892	-0.54	0.588	reproduction	1.0142	0.4058	2.499	0.012 *
log(Lysozyme)	Intercept	1.10660	0.07658	14.451	<0.001 ***	Intercept	-1.07896	0.19550	-5.519	<0.001 ***
	status	0.14684	0.12329	1.191	0.234	status	-0.47254	0.50454	-0.937	0.349
	sex	-0.06550	0.08409	-0.779	0.436	sex	-0.06985	0.26523	-0.263	0.792
	reproduction	0.01037	0.11745	0.088	0.930	reproduction	-0.83307	0.42374	-1.966	0.049 *
Haemagglutination	Intercept	7.2516	0.2150	33.73	<0.001 ***	Intercept	0.9904	0.2154	4.598	<0.001 ***
	status	-0.5547	0.2852	-1.94	0.052	status	-0.2289	0.4999	-0.458	0.647
	sex	-0.4036	0.2209	-1.83	0.068	sex	-0.6460	0.2663	-2.426	0.015 *
	reproduction	-0.2879	0.2636	-1.09	0.275	reproduction	-0.8855	0.4141	-2.139	0.033 *
Haemolysis	Intercept	6.15860	0.18726	32.89	<0.001 ***	Intercept	0.5651	0.2163	2.613	0.009 **
	status	-0.74563	0.31864	-2.34	0.019 *	status	-0.4127	0.5016	-0.823	0.410
	sex	-0.03002	0.20661	-0.15	0.885	sex	-0.2172	0.2665	-0.815	0.415
	reproduction	-0.40895	0.29183	-1.40	0.161	reproduction	-0.4791	0.4124	-1.162	0.245

Table S3: Summary output of the GLMMs to test for differences in the mean and variance of “high cost” immune effectors.

Effect on the mean						Effect on the variance				
**Response**	**Predictor (fixed effects)**	**Estimate**	**Std. Error**	**z**	**p**		**Estimate**	**Std. Error**	**z**	**p**
Total leucocytes	Intercept	84.599	6.426	13.165	<0.001 ***	Intercept	6.2512	0.2150	29.080	<0.001 ***
	status	-9.216	12.229	-0.754	0.451	status	-0.3496	0.6657	-0.525	0.599
	sex	-11.149	4.047	-2.755	0.006 **	sex	-0.1370	0.2751	-0.498	0.618
	reproduction	16.283	9.000	1.809	0.070	reproduction	0.2678	0.5984	0.448	0.655
haptoglobin binomial	Intercept	1.7129	0.4198	4.080	<0.001 ***					
	status	-2.0296	0.8368	-2.425	0.015 *					
	sex	-0.9708	0.4681	-2.074	0.038 *					
	reproduction	0.2173	0.7166	0.303	0.762					

Table S4: Summary output of the GLMMs to test for differences in the mean and variance of the differential white blood cells

Effect on the mean						Effect on the variance				
**Response**	**Predictor (fixed effects)**	**Estimate**	**Std. Error**	**z**	**p**		**Estimate**	**Std. Error**	**z**	**p**
basophil granulocytes	Intercept	2.60467	0.35342	7.370	<0.001 ***	Intercept	1.8625	0.1890	9.852	<0.001 ***
	status	0.11607	0.76019	0.153	0.879	status	-0.8225	0.4786	-1.719	0.086
	sex	-0.54869	0.40609	-1.351	0.177	sex	-0.3952	0.2847	-1.388	0.165
	reproduction	-0.02087	0.69358	-0.030	0.976	reproduction	0.2770	0.4241	0.653	0.514
eosinophil granulocytes	Intercept	12.3976	1.9987	6.203	<0.001 ***	Intercept	5.27833	0.21126	24.985	<0.001 ***
	status	23.1386	4.3982	5.261	<0.001 ***	status	-0.03524	0.49282	-0.072	0.943
	sex	-0.4623	2.4184	-0.191	0.848	sex	-0.07766	0.25839	-0.301	0.764
	reproduction	1.0329	3.6249	0.285	0.776	reproduction	-0.11356	0.39515	-0.287	0.774
sqrt(heterophil granulocytes)	Intercept	3.9517	0.2044	19.334	<0.001 ***	Intercept	0.8234	0.1965	4.190	<0.001 ***
	status	-1.4923	0.4683	-3.186	0.001 **	status	-1.2678	0.4797	-2.643	0.008 **
	sex	-0.1791	0.2297	-0.780	0.436	sex	-0.3577	0.2530	-1.414	0.157
	reproduction	0.1985	0.4433	0.448	0.654	reproduction	0.4104	0.3965	1.035	0.301
lymphocytes	Intercept	38.600	3.766	10.251	<0.001 ***	Intercept	5.2269	0.2151	24.304	<0.001 ***
	status	-25.514	8.600	-2.967	0.003 **	status	-1.3770	0.6209	-2.218	0.027 *
	sex	-7.542	2.340	-3.223	0.001 **	sex	-0.2644	0.2847	-0.929	0.353
	reproduction	12.683	6.100	2.079	0.038 *	reproduction	0.8547	0.5398	1.583	0.113
log(monocytes)	Intercept	2.14543	0.20510	10.461	<0.001 ***	Intercept	-0.55146	0.22282	-2.475	0.013 *
	status	0.70363	0.44294	1.589	0.112	status	-1.00727	0.51500	-1.956	0.051
	sex	-0.02772	0.13573	-0.204	0.838	sex	0.23409	0.26990	0.867	0.386
	reproduction	0.14235	0.25529	0.558	0.577	reproduction	0.05926	0.40305	0.147	0.883

Table S5: Methods of the parasite screening and serology of Egyptian geese from Namibia and Germany.

**Parasitology:**	Group	Transmission mode	Method	Material
	Ectoparasites	direct	morphology	carcasses
	Euhirundidae	direct	morphology	carcasses
	Intestinal helminthes		morphology	carcasses
	Cestoda	vector	morphology	carcasses
	Nematoda	direct	morphology	carcasses
	Trematoda	vector	morphology	carcasses
	Haematozoa	vector	morphology	blood smear
**Bacteria:**	Target genes	Transmission mode	Method	Material
	Riemerella anatipestifer 16S rRNA gene	direct	PCR	pharyngeal swab
	Mycoplasma spp. 16S rRNA gene	direct	PCR	pharyngeal swab
**Serology:**	Antigen	Transmission mode	Method	Material
	IAV	direct	ELISA	serum
	AAvV-1	direct	ELISA	serum
	WNV	vector	ELISA	serum

Abbreviations: IAV= Influenza A virus; AAvV-1= *Avian avulavirus 1*; WNV= *West Nile virus*; ELISA= Enzyme linked immunosorbent assay; PCR= Polymerase chain reaction
